# The benefits of multidisciplinary collaboration in the successful management of two complex feline cases

**DOI:** 10.1111/avj.70083

**Published:** 2026-04-21

**Authors:** BE Bollaert, JM Ley, HE Zulch

**Affiliations:** ^1^ Healthy Pet Behaviour Services Brisbane Queensland Australia; ^2^ Melbourne Veterinary Specialist Centre Glen Waverley Victoria Australia; ^3^ School of Life Sciences University of Lincoln Lincoln UK

**Keywords:** animal welfare, chronic, complex, multidisciplinary team approach, patient outcomes, veterinary teams

## Abstract

Despite a frequent misconception that behavioural concerns are diagnoses of exclusion, medical conditions and behavioural disorders are frequently inter‐related. In human medicine, the ‘multidisciplinary team’ approach (MDTA) is considered the most efficacious way of managing patients with complex and chronic health conditions. The case series illustrates how the MDTA for complex veterinary cases improves diagnosis, treatment and management for positive long‐term outcomes in feline patients' health and welfare. Feline patient 1, a 2‐year‐old spayed domestic shorthair cat, presented for repeated episodes of self‐trauma to her face and other areas. Detailed diagnostics explored dermatological, behavioural and neurological differential diagnoses. No single diagnosis could completely explain the feline patient's clinical signs or behaviour. A treatment plan involving medication, environmental management and behaviour modification was implemented to improve her health and welfare. Feline patient 2, a 3‐year‐old spayed domestic long‐haired cat, presented after surgical removal of a trichobezoar (second surgery). The diagnostic work‐up considered dermatological, behavioural and gastrointestinal causes. A working diagnosis was made of anxiety disorder with possible compounding factors of gastrointestinal and dermatological conditions. The treatment plan included medication, environmental management and behaviour modification. The overgrooming and fur ingestion behaviours resolved, and long‐term strategies maintained physical and emotional health and welfare. Without the MDTA, neither feline patient would have received effective care. Using the MDTA for managing complex veterinary cases provides skilful assessment in determining aetiologies, treatment of complex conditions, and identification and management of contributing factors, which may affect and predict flare‐ups. The MDTA improves outcomes for owners, feline patients and the veterinary teams who care for them.

**Abbreviation**
MDTA: multidisciplinary team approach

## Practitioner Points

- Medical conditions, including those involving mental and emotional problems, influence animals' cognitive and behavioural outputs and their long‐term health.
- The multidisciplinary team approach (MDTA) assists veterinary professionals in recognising and addressing influences on animal illness and wellness, especially conditions that are complex and chronic.
- Effective implementation of the MDTA illustrates how long‐term positive welfare outcomes can be achieved for feline patients, carers and clinicians.

As advances in veterinary medicine have enabled veterinarians to provide diagnoses and treatments for more complex and chronic health conditions, the increased numbers of pets with these conditions creates additional challenges for the profession. This means that, as in human medicine, the need for new approaches to patient management has arisen. This is particularly the case as complex health conditions are often multifactorial in development, with biological, psychological, emotional, environmental and social factors (and their interactions) playing a role.^1^, ^2^ In human medicine, the multidisciplinary team approach (MDTA) is regarded as the most efficacious way of managing patients with complex and chronic health conditions as no single, specialised health professional can deliver all of the required care.^3^ This approach may be a useful model to the veterinary profession. There are many potential benefits of interprofessional collaboration to patients, their owners and the individuals within the veterinary team as well as the organisations within which they work.^4^, ^5^ Additionally, sometimes limited resources are more efficiently utilised when a multidisciplinary approach is used.^6^, ^7^

A particular area where a MDTA is of value is where there may be an inter‐relationship between behavioural presentations and physical illness.^8^, ^9^ There is a frequent misconception that behavioural concerns are diagnoses of exclusion. Stated differently, a physical presentation is frequently attributed to behavioural maladaptation solely after the exclusion of underlying physical disease. In reality, an animal's behaviour and physical health are interrelated.^10^

The two cases presented in this report aim to illustrate how multidisciplinary collaboration and understanding of treatment of the feline patient as a whole, integrated individual is essential in improving the quality of life for feline patients with complex chronic health conditions, as well as their owners. Consent was given by the owners of the cats for the publication of their data.

## Feline patient 1

### Presentation

Feline patient 1 was an estimated 3‐year‐old female spayed domestic short hair cat when referred to a behavioural referral service for recurrent episodes of self‐inflicted alopecia and trauma to multiple body parts. At presentation to the behavioural service, she weighed 5.8 kg.

### History and presenting signs

Feline patient 1 and her sibling were surrendered to a welfare organisation at around 12 months of age. Both cats were in poor general health. Feline patient 1 had an amputated tail tip, a small wound near the base of her right ear and bilateral yeast otitis externa which responded well to off‐label use of terbinafine, florfenicol and betamethasone acetate aural gel (Osurnia®, 1.2 g/tube, dosed once; Dechra, Somersby, NSW). The tail and ear base wounds began healing, so no further treatment was deemed necessary at that time. However, over the following weeks she developed recurrent patches of alopecia and self‐trauma to her tail, face, axillae and caudal thighs. The tail required more proximal amputation, whereafter, with strict management including the use of an Elizabethan collar, it healed well.

To mitigate potential shelter environment stressors causing overgrooming and self‐trauma, feline patient 1 was fostered into a multi‐cat home but was moved back to the shelter due to intercat aggression. A new foster home was found where she lived with one dog and three cats. The self‐induced alopecia and trauma episodes (some of which were witnessed by the carer) continued throughout this time despite feline patient 1 receiving additional diagnostic and treatment interventions (See Table 1 below). Additionally, feline patient 1 was started on Gabapentin (Neurontin®, ½ × 100 mg capsule ~50 mg, orally, twice daily; Viatris, Millers Point, NSW) and Clomipramine (Clomav®, ¾ × 5 mg tablet ~3.75 mg, orally, once daily; Mavlab, Logan City, QLD). Initially, feline patient 1 responded well with episodes of self‐trauma decreasing in frequency, but progress plateaued after 18 months. She was then referred to the behavioural referral service. Diagnostic workup and treatment trials prior to behavioural assessment are summarised in Table 1 below.

**Table 1:** Feline patient 1: Diagnostic tests and treatment trials prior to behaviour assessment

Reason	Test	Outcome
Ectoparasites	Clinical exam with no evidence of fleas and flea dirt Treatment trial with fluralaner and moxidectin (Bravecto plus®; MSD Animal Health; Macquarie Park, NSW)	No clinical evidence found No response
Possible dermatological and gastrointestinal allergy/atopy/intolerance/discomfort/pain	Dietary trials (6–8 weeks): Delicate care cat sensitive skin or stomach® (NB Petfoods, North Fremantle, WA) Royal Canin Anallergenic® (Royal Canin, Docklands, VIC) Prime 100 Rosemary and Lamb Dog Roll® (Prime 100 Pty Ltd, Derrimut, VIC) Histopathology: Punch biopsies from affected skin Treatment trials: Several courses of prednisolone^a^ (2.5 mg, orally, twice daily initially & tapered gradually) Oclacitinib maleate (Apoquel®; Zoetis, Silverwater, NSW; 2.7 mg twice daily initially and tapered gradually)	No response to any dietary changes No abnormalities reported No response and unable to prevent recurrence
Infectious causes of skin lesions	Culture of punch biopsy samples from affected skin Subsequent purulent infection of punch biopsy sites and self‐trauma sites Rx with cephalexin^a^ (100 mg, orally, twice daily) and cefovecin maleate (Convenia®; Zoetis, Silverwater, NSW; injectable, once)	No microbes cultured Infections cleared with standard course; frequency or intensity of self‐trauma episodes remained the same.
Possible underlying nasal sinus or dental disease	Radiography	No abnormalities found
Possible underlying metabolic or other organ system disease	Full panel Haematology and biochemistry panel	No abnormalities detected
Possible systemic disease	FIV/FeLV snap test	Negative
Possible systemic disease	Urine specific gravity Urinalysis	>1.050 No abnormalities detected

^a^ Particular brand used and specific dose rates are unknown as related to historical clinical notes.

### Behaviour assessment

Feline patient 1 presented (wearing an Elizabethan collar) as a calm, relaxed and bold cat. She readily came out of her carrier, approached the clinician to greet, explored the consulting room and engaged with puzzle feeders and toys. It was noted that she had disproportionately short legs for her body length and had mild excoriations in the periaural areas dorsal to her eyes, mild dental disease and a body condition score of 6/9. During a gentle physical exam and postural manipulation, several behavioural and neurological abnormalities were noted (see Table 2).

**Table 2:** Feline patient 1: Behavioural and neurological abnormalities noted during the behaviour assessment

Sought intense tactile interactions (firm, even rough rubbing and petting) with carer, clinician and student	Intensity abnormal; typically, would have resulted in self‐protective response; the carer reported that intensity would induce self‐protective behaviours from her other pets
Proprioceptive deficits	Limbs regularly crossed the midline when walking; when stationary, limbs were left in abducted positions with apparent decreased awareness of limb positioning; occasional knuckling and stumbling were observed; this was reported to be common at home
Kitten‐like pattern of climbing	Extended forelimbs, grabbed with claws and pulled body up rather than jumping
Absent/delayed righting reflex	During petting episodes rolled onto side and back, and off person's lap; common at home and occasionally landed on her side/back without righting. No startle/distress signs
Undershooting when jumping	Estimation of distance inaccurate with falling short; no startle/distress
Clumsiness and ataxia	Limbs did not always advance in the direction in which she intended to travel; no startle/distress when she walked into or distal body parts bumped into items.
Reduced startle response	No startle to loud claps behind her, but able to respond to carer calling her, the sound of treat bag opening, lure toy with bell behind her and out of visual field.

Behaviourally, feline patient 1's carer noted she approached the companion cats with prosocial, relaxed behaviour but struggled to respond to their social cues, for example, she did not end the interaction when they attempted to (turned or moved away). Her carer was generally able to interrupt and redirect her attention (called her, tossed a treat or engaged in play) but could not always intervene in time. If the other cat hissed, growled or swatted at feline patient 1, she startled mildly, moved away briefly and returned to interacting without any obvious signs of distress or fear. Hence, feline patient 1 was separated from the other pets when her carer was unable to supervise interactions. Her carer felt that her medications had helped a little with her social interactions and that the Elizabethan collar was preventing retraumatisation of her skin.

### Provisional behavioural diagnosis and treatment plan

An assessment was made of an abnormal repetitive behaviour (ARB) displayed as intense face rubbing and grooming of toys and people with compounding factors of proprioceptive deficits; with possible sensory processing or pain processing disorders, and potential underlying gastrointestinal issues not yet investigated with biopsy. ARBs are defined as a group of heterogeneous behaviours that are described as repetitive, invariant, occurring out of context and appearing to be purposeless or functionless, and may include stereotypies and compulsive behaviours.^11^ Many ARBs may have underlying neurobiological and physical pathophysiology.^11^ The approach to treating ARBs should focus on the underlying cause and may require therapeutic response trials.^11^ Clomipramine and fluoxetine are considered suitable treatment options if the ultimate diagnosis is of a compulsive or conflict‐induced behavioural condition; however, additional medications may be required if there are also components of pain, neuropathic pain or other physical conditions.^11^ This was the clinical approach used in both cases. The initial treatment plan (see Table 3) focussed on supporting and addressing these factors.

**Table 3:** Initial treatment plan for feline patient 1

Medication	Management	Behaviour modification exercises
Clomipramine increased to 5 mg sid ~0.9 mg/kg (Clomav®, 1 × 5 mg tablet, orally, once daily; Mavlab, Logan City, QLD) Gabapentin 50 mg ~9 mg/kg, orally, twice daily; additional 50 mg orally ~17 mg/kg, 90 min prior to stressful situations (Neurontin®, ½ × 100 mg capsule ~50 mg; Viatris, millers point, NSW) Omeprazole 5 mg ~0.9 mg/kg orally, once daily, 1 hr before meals (omeprazole Sandoz®, ¼ × 20 mg tablet ~5 mg; Sandoz, Macquarie Park NSW)	Elizabethan collar when carer was unable to supervise Separation from companion pets when owner was unable to supervise Continue with predictable routines Continued provision of opportunities to play Physical barriers to prevent access to problem areas, e.g., Christmas tree	Reinforcement of calm and relaxed behaviours Increase reliability of recall (to interrupt unwanted interactions) Use a positive interrupter (call pet's name in a happy voice) and redirect undesirable behaviours (e.g., face rubbing, persisting with social interactions)

The feline patient was referred to a Board‐Certified Veterinary Neurologist for evaluation of neurological signs.

### Neurological assessment

Feline patient 1 was bright, alert and responsive. She displayed hypertonic forelimb gait, mild generalised ataxia, intermittent crossing of thoracic limbs, occasional spontaneous knuckling on hindlimbs and subtle intermittent titubation and head bob/tremor. She showed decreased pelvic limb awareness but intact handstand and weight bearing. Extensor Postural Thrust was slower on her left hindlimb than the right. Pelvic limb hopping proprioception was mildly delayed. She did not permit passive proprioceptive repositioning. Spinal reflexes were intact with normal muscle bulk and tone. Cranial nerves were intact, as was continence. No spinal hyperpathia observed; nociception and eye exams unassessed.

Suspected neuroanatomical localisation:

- C1–C5 OR Cerebellar

Differential neurological diagnoses:

1. Anomalous – cerebellar abiotrophy/hypoplasia versus C1–C5 vertebral body malformation.
2. Infectious/inflammatory – meningitis/meningomyelitis (i.e., immune‐mediated vs. bacterial vs. viral) (ruled out as the patient was stable and condition not currently progressing).
3. Metabolic – hypocalcaemia (Ruled out by biochemistry – total calcium and albumin within normal limits).

As further assessment (brain +/− cervical‐spine MRI +/− CSF analysis) was unlikely to change the treatment plan, the owner and clinician decided to continue treatment without further diagnostics.

### Final neurological diagnosis

A congenital anomaly causing a range of sensory and motor deficiencies. Feline patient 1 was referred back to the behavioural service for ongoing care. No neurological‐specific treatment was indicated.

### Treatment

The treatment plan (see Table 4) focused on preventing self‐trauma by addressing and preventing factors which could perpetuate incidents and implementing interventions to promote a good quality of life for feline patient 1, her carer and her companion animals.

**Table 4:** Ongoing treatment plan for management of patient 1's behavioural and neurological problems

Medication	Management	Behaviour modification
Clomipramine 5 mg, orally, once daily Gabapentin 50 mg, orally, twice daily; 100 mg, orally, 90 min prior to stressful situations Omeprazole discontinued – no response	Continued use of Elizabethan collar when unsupervised Continue to supervise daily periods without Elizabethan collar to allow grooming and ease of movement Continue with predictable routines Continued separation from other pets when unsupervised to prevent escalation of unwanted interactions Continued prevention of access to problem areas e.g. Christmas tree Continued provision of access to suitable latrines, food and water stations, resting and sleeping spaces, exercise and play and opportunities for appropriate grooming and social interaction.	Continued use of positive interrupter and redirection away from rubbing or grooming Continued use of positive interrupter and redirection away from other pets wanting to end the interaction Continued reinforcement of calm and relaxed behaviours Continued reinforcement of recall Place training (teaching a pet to go to a particular location on cue) to facilitate calm and predictable routines for play and mealtimes

*Note*: The carer continued to monitor frequency and intensity of grooming and rubbing behaviours, social interactions and the patient's wellbeing by diary and video. This facilitated evaluation of progress.

### Follow‐up

After 8 months on Clomipramine (5 mg, orally, once daily), it was unclear whether the medication was facilitating improvement or whether management and behaviour modification were effective on their own. Clomipramine was reduced to 2.5 mg once daily to assess whether progress was able to be maintained. After 2 weeks, feline patient 1 was restless and had started charging at and cornering the other cats in the household. She was agitated when wearing the Elizabethan collar. She removed the collar, something she had not previously done, and one episode resulted in severe self‐trauma to her right foreleg. Clomipramine dose was returned to 5 mg once daily; Gabapentin dosage was increased to 100 mg twice daily for approximately 4 days, then reduced once again to 50 mg twice daily. Within 2 weeks she was more settled, interactions with companion cats had calmed, she was able to rest and relax, and was no longer agitated or trying to remove her collar. She remained stable on the above regimen at her follow‐up 6 months later when she was discharged from the behaviour service.

## Feline patient 2

### Presentation

Feline patient 2, a 3‐year, 7‐month‐old female spayed domestic medium hair cat, was referred to the behavioural service by a small animal surgical registrar following recent surgery for removal of a small intestinal foreign body. She was examined by the referring surgeon immediately prior to the behaviour assessment and was found to be in good physical health. At presentation to the behavioural service, she weighed 4.5 kg.

### History and presenting signs

In the past 12 months, feline patient 2 had undergone two exploratory laparotomy surgeries. Both involved removal of obstructive hairballs from the gastrointestinal tract. On ultrasonography prior to the second surgery, fluid and gas‐dilated stomach, duodenum and jejunum orad to a long shadowing hyperechoic object consistent with mechanical obstruction/ileus, peritoneal effusion, thickened gallbladder wall and dilated bile ducts were noted. These resolved after the obstruction was removed via gastrotomy after milking the soft pliable foreign body orad from the small intestine. Feline patient 2 recovered uneventfully from the second surgery. At the post‐operative follow‐up with the surgeon, the carer reported that feline patient 2 groomed herself excessively during time alone at home, or when visitors were present. The surgeon referred her to the behavioural referral service for assessment.

### Behaviour assessment

The telemedicine assessment was run via a video conferencing platform (Zoom Communications®). This minimised the fear and distress that patient 2 experienced during car rides and around unfamiliar people, and permitted a real‐time visualisation of her behaviour at home.

The carer reported that the highest intensity grooming, plucking and ingestion of fur occurred with visitors to the home and when her parents looked after feline patient 2. Her carer found large tufts of fur on the floor, bald patches on the feline patient in variable locations, and frequent furball vomits during and after these times. The carer's parents reported that feline patient 2 hid while they were awake and that she only came out to eat and drink when they were asleep in bed, evidenced by food and water levels changing overnight.

Feline patient 2 also plucked and ingested fur when home alone (confirmed via video footage). These behaviours reportedly intensified at other times as well (e.g., lawnmower or vacuum cleaner use; significant neighbourhood noise and activity).

Patient 2 first started vomiting up fur balls around 18–24 months prior, when they moved into their current home. Previously, she had only spent short periods at home alone as a result of work schedules and Covid work from home public health rules. Apart from plucking fur, the carer had noticed that other grooming behaviours (licking down her back, flank and towards her tail base) had also increased in frequency and intensity over the preceding several months. She vomited furballs every 2–3 days, more frequently during times when grooming and plucking were more intense. This occurred despite being on Hill's Science Diet™ Adult Hairball Control, praziquantel (Popantel® Tapeworm tablets; Zoetis, Silverwater, NSW; 50 mg, orally, 3 monthly) and fluralaner & moxidectin topically (Bravecto® Plus for Cats; MSD Animal Health; Macquarie Park, NSW; 40–94 mg fluralaner/kg and 2–4.7 moxidectin/kg).

The patient exhibited pronounced fear in the car (crying, yowling, pacing) and at the vet (hiding in the carrier, remaining immobile on the examination table). She also paced the inside of the home watching out of the windows for activity at the front of the house, sometimes growling at people walking past on the foot path (~10–15 m away).

Her carer reported that she would unpredictably scratch her when resisting or rejecting touch. She also reported that play sometimes escalated to rough scratching and biting, and that if she was already in an agitated mood (high arousal, flicking tail and crouched posture), she often rushed out and scratched and bit her carer's feet and ankles, even if they were not interacting at the time. Occasionally, her carer found it difficult to recall her indoors, and picking her up at these times had previously resulted in scratches and nips to the carer.

Although a clinical examination was not performed during the assessment, video showed that feline patient 2 had a sparse and patchy coat on her flanks and abdomen, with a neatly clipped abdominal area from her recent surgery. Her body condition score was 5/9, with no other physical abnormalities observed.

Possible differential diagnoses included fear of a range of primarily social, but some non‐social stimuli, in some instances with frustration secondarily (as a result of inability to escape the stimulus, as when visitors were present); frustration due to perceived lack of autonomy and/or lack of access to desired resources; conflicted emotions – all of these leading to overgrooming as an attempted coping strategy; potential musculoskeletal, gastrointestinal and/or dermatological discomfort or pain. Full haematology and biochemistry panels (including Total T4) were recommended, along with further investigation of possible gastrointestinal and dermatological issues. Videos of the patient's movement did not show any gait or postural abnormalities, so musculoskeletal issues were considered less likely. Patient 2's carer was reluctant to undertake further diagnostic tests at this stage as she felt these would cause further distress and potentially exacerbate the overgrooming behaviour, but was open to pursuing these if no improvement occurred by the time Patient 2 was seen for behavioural follow‐up.

An assessment was made of frustration associated with time alone evidenced by overgrooming, fur plucking and fur ingestion; frustration and potential discomfort related to interactions with carer (scratches and bites during interactions); fear of unfamiliar people (avoidance, hiding) and frustration associated with their presence in the home (overgrooming and hair ingestion); anxious behaviours during changes in routine (pacing, hiding, seeking close contact with her carer).

### Treatment

The treatment plan (see Table 5) focused on reducing intense grooming, fur plucking and ingestion, and scratches and bites to her owner; addressing and preventing situations that would precipitate or perpetuate the problem behaviours; increasing the use of protective factors which helped maintain good quality of life for feline patient 2 and her carer.

**Table 5:** Treatment plan for management of patient 2's behavioural problems

Medication	Management	Behaviour modification
Fluoxetine 5 mg ~1.1 mg/kg (Zactin®, ¼ × 20 mg tablet; orally, once daily; Viatris, Millers Point, NSW.) Pregabalin ~8 mg ~1.7 mg/kg (apo‐pregabalin® 1/3 × 25 mg capsule; Arrotex, Cremorne, VIC) orally, once daily; 12.5 mg ~2.8 mg/kg (~1/2 × 25 mg capsule) orally, 90 min prior to stressful situations Paraffin 400 mg/g palatable paste (Troy Laxapet gel; Troy, Glendenning, NSW) orally, 2 cm twice weekly for 2 weeks then once weekly to fortnightly thereafter Synthetic analogue of the F3 feline facial pheromone diffuser (Feliway® classic diffusers; Ceva, Glenorie, NSW) diffusers throughout the home and in accordance with the manufacturer's instructions	Calm and predictable daily routine Calm and predictable carer departure and arrival routines, pairing departure with an enjoyable activity Auditory and visual screening: Frosting to windows that look onto the footpath; relaxing music when carer was out and at busier times in the neighbourhood Structured play times with a variety of toys; no hands or feet during play; sessions ending with opportunities for independent play Food, water and litter trays moved to locations away from visitor areas Visitors not to enter patient 2's safe space (owner's bedroom and en suite) Carer to pay attention to subtle body language when feline patient 2 was avoiding or ending contact, and cease interacting Use toys/treat scatter to redirect patient 2 away from carer when patient is emotionally aroused	Positive reinforcement of spontaneously calm behaviours ‘Mat settling’ where relaxation is paired with a mat that the patient settled on ‘Touch’ cue to facilitate movement onto or off furniture ‘Touch’ sequence of repeating nose targeting to improve client–patient communication and redirection ‘Inside’ cue to facilitate returning indoors and pairing with a high value reinforcer, e.g., treat or play

Counterconditioning and desensitisation to visitors, carrier training, calm vet visits and pet sitting plans were also discussed as future steps. Progress was tracked using a diary and video logs.

### Follow‐up

At the 6‐week follow‐up, patient 2 had no further vomiting, groomed at a normal frequency and seemed more relaxed and content. However, she had still been distressed when the garden service and cleaner came, and when she went for her vaccination appointment at the general practice vet. She had not had the higher dose of Pregabalin on these days. Despite being distressed, she recovered more quickly and no fur plucking or ingesting occurred. Her carer wanted to reduce the frequency of pregabalin dosing to an as needed basis as patient 2 had shown improvement and she was concerned that she did not seem as playful as previously. She tapered the pregabalin dose over the following 2 weeks and only dosed prn. However, within a few days, grooming frequency increased and two small clumps of hair were found after returning home; patient 2 had tried to bite and scratch when her carer petted her; she seemed more on edge and agitated generally. On restarting the daily dose of Pregabalin ~8 mg these behaviours subsided again. Patient 2 has remained stable on Fluoxetine 5 mg sid, Pregabalin 8–12.5 mg in AM; additional as needed 8 mg dose 90 min prior to visitors in the evening. She has continued behaviour modification and management strategies.

Four months post‐assessment, patient 2 engaged in regular play, showed calm behaviours during interactions with her carer, was more relaxed during the day and had recently started coming out to greet and interact calmly with visitors. Although the carer did not realise or report a disrupted sleep pattern during the initial assessment, despite the clinician enquiring specifically about sleep patterns, quality and quantity, her carer reported at follow‐up that patient 2 had started sleeping more soundly overnight. Her carer was very happy that patient 2 was now able to live a calmer and relaxed life and that the underlying cause for the life‐threatening hair ingestion and obstruction was being addressed. No further diagnostic assessments were undertaken.

## Discussion

Both patients in these cases required a multidisciplinary approach for appropriate diagnoses and treatment. In case 1 the multidisciplinary team included the general practitioners and GP nursing team; a veterinary behavioural medicine resident (the first author), behaviour nurse and trainer; and a registered specialist veterinary neurologist and neurology resident. In case two the multidisciplinary team included the surgical registrar and specialist surgical nursing team; a veterinary behavioural medicine resident (the first author), and behaviour nurse and trainer. In addition, both cases were also discussed with two registered specialists in veterinary behavioural medicine (the co‐authors). The need for a multidisciplinary approach in managing complex veterinary cases has been discussed in a recent retrospective study of 38 cats with biopsy‐confirmed oral eosinophilic disease. Where concurrent gastrointestinal, skin‐related and respiratory signs were present, and comorbidities included periodontal disease and mandibular lymphadenomegaly, these cats showed an increased likelihood of developing oronasal fistulae.^12^ In a case report on a dog with a congenital extra‐hepatic portosystemic shunt, displaying physical signs such as polyuria and polydipsia, behavioural signs of aggression towards the caregivers and anxiety were also noted. Once again, a multidisciplinary approach was successful in improving the dog's clinical signs, quality of life and in reducing aggressive behaviours.^13^ A retrospective review found that a significant number of dogs and cats with chronic enteropathies (confirmed by histopathology) showed pica, as a clinical sign.^14^ The authors suggested that when patients, especially adult animals, present with pica, alongside surgical removal of foreign bodies, they should be medically investigated in more detail to assess the presence of underlying disease. A growing body of literature^9^, ^15^, ^16^, ^17^, ^18^, ^19^, ^20^ shows the association between medical conditions, including those involving mental and emotional problems, and animals’ cognitive and behavioural outputs, and long‐term health. This underlines the fact that the brain and the rest of the body are integrated and influence each other, rather than separate entities housed together but functioning independently. These studies, together with the cases presented in this report illustrate how multidisciplinary collaboration between veterinary professionals across the realms of physical, mental and emotional domains is necessary for effective treatment and management.

In human health it is being recognised that the pathogenic mechanisms involved in illness do not divide neatly between physical health conditions and mental health conditions, and that epidemiological evidence in chronic health issues involves biological, psychological, social and environmental factors.^21^ This has led to human medical professionals recognising that many chronic and complex cases require expertise from a number of different fields^3^ in order to address the patient as a whole individual, within their social and physical environment. However, medical professionals still find it challenging to integrate physical disease with psychological and social health or illness^22^; for example, Xiao and colleagues reported that medical staff in the psychiatric department paid more attention to human patients' psychosocial status than staff in non‐psychiatric departments. They also found that patients believed that medical staff refused to attend to and did not encourage reporting of any information other than disease. This suggests that those who do not pay attention to lifestyle factors and patient's psychological and social health may be missing out on important and relevant factors in addressing predisposing, precipitating, and perpetuating factors in their patients’ chronic health conditions.^21^ It may also suggest that these clinicians are under severe time pressure and/ or may feel ill‐equipped or trained to deal with these components of addressing and managing their patients’ health care.^22^ In addition, when the focus is on mental health, the opposite may be true. A study reporting on human mental health clinicians (psychiatrists, psychologists, social workers and nurses) found that although physical health was raised in the majority of intake visits, and discussed substantially in over half, clinicians who do not inquire about physical illness may also be missing it.^23^ The study discussed how these undiagnosed or overlooked illnesses may affect the psychotherapeutic process, negatively affecting patients’ mental health, resulting in exclusion of relevant differential diagnoses, and missed opportunities for preventing comorbid conditions.

Translating this to the veterinary sphere, it is essential that those working in the animal behaviour field are highly qualified, skilled and proactive in recognising the relationship between physical and behavioural health and in advising clients on seeking further treatment for all concurrent medical conditions.

Additionally, it highlights the important role that General Practice and Specialist Veterinarians in disciplines other than behaviour; Veterinary Nurses Technicians and Physiotherapists; and other allied Animal Professionals play in referring patients to appropriately qualified veterinary professionals for behavioural concerns, just as they would for other physical health concerns.

In the two cases discussed, had the clinicians involved not paid attention to the biological, psychological, social and environmental components affecting the overall health of these patients, they would not have received appropriate and timely care. Neither would the patients have been managed well by their carers over the longer term, which could have resulted in disruption to the human‐animal bond and deterioration in welfare for both the patient and carer.

Collaboration between professionals in treating these two patients resulted in sharing of the cognitive burden associated with diagnosing and treating these complex and chronic health conditions. It is well recognised that this burden is a significant factor affecting veterinary professionals currently.^24^, ^25^ Communicating effectively with carers, referring appropriately and discussing cases as a multidisciplinary team not only improved the outcomes for these two patients, but also their carers and the veterinary staff involved.

The carers involved in these two cases were very appreciative of the time taken to discuss, investigate and collaborate with other professionals regarding their animals' care. Carers with pets that have behavioural problems^26^ or chronic or terminal diseases experience significant burden, stress, symptoms of depression/anxiety and impaired quality of life^27^ and financial well‐being.^28^ Burden of care is also a significant factor affecting client adherence to treatment plans in animals with chronic or terminal disease.^29^ It is recognised that empathetic discussion and explanations of pets' conditions, why pets may or may not respond to particular treatments as well as a clear understanding of the factors that affect response, are important for carers to make informed decisions about their pets' care.^30^ Supporting the client's sense of control and the veterinarian demonstrating that they understand the client's care burden enhances communication and alleviates that burden even when amelioration of the animal's presentation is not feasible.^31^ This was achieved in these cases through clear communication between the clinicians and the carers and between the clinicians themselves. This allowed the clients to better understand their pets’ clinical signs, diagnosed conditions and treatment plans so that they had an appreciation of how the biological, psychological, social and environmental factors would affect their animals’ overall health. The carers of the patients discussed in this case report both verbalised satisfaction that their concerns had been addressed whilst understanding that their animals had chronic and complicated conditions that would require ongoing care, may experience acute exacerbations/flares, and would never be ‘cured’.

In human medicine, a framework for developing effective multidisciplinary collaboration needs to take several factors into consideration.^32^ These include expanding roles for medical assistants; adding new team members (physician assistants, nurse practitioners, pharmacists or behavioural health providers); providing physical spaces that facilitate interaction; promoting cognitive factors that encourage shared purpose and goals; effective communication; leadership structures that reflect the team goals; capacity for team members to effect change and modify protocols where appropriate and having a team with skills suited to the geographical and social context of the practice. While this is no small task, we suggest that the veterinary industry can start to move towards this. In the authors' experience, some practical ways in which this can be achieved include ensuring that behavioural medicine is appropriately covered at undergraduate study and widely available as continuing professional development; facilitating the development of professional networks across disciplines; including Behaviour as a specialism in referral practices and hospitals; developing multidisciplinary ‘Grand Rounds’ and discussion groups in larger hospitals and between practices; involving, training and delegating appropriate tasks to clinical and paraclinical support staff; ‘cross‐pollination’ of ideas between different specialties at professional conferences; clinical rotations for under‐ and post‐graduates in clinical behavioural medicine practices; and through cross‐referral and case discussions of individual cases. This is a vital area of professional development and research that has many potential benefits.

## Conclusion

There is substantial scope for improvement in multidisciplinary collaboration and the importance of recognising and addressing all influences on animal illness and wellness, both physical and behavioural. It is important for veterinary science as a field, and veterinary professionals as individuals, to recognise this in order to maximise effective long‐term care for animal patients. In so doing, a related benefit of improved mental health for the veterinary professionals involved may also be achieved.

Whilst by their very nature, complex cases can be difficult to manage, these two cases have illustrated how long‐term positive welfare outcomes can be achieved for patients, carers and clinicians through multidisciplinary collaboration.

## Conflicts of interest and sources of funding

The authors have declared that they have no conflicts of interest.

## Data Availability

Data sharing is not applicable to this article as no new data were created or analysed in this study. Consent was given by the owners of the cats for the publication of their data.
